# Updating Insights into the Catalytic Domain Properties of Plant *Cellulose synthase *(*CesA*)** and *Cellulose synthase-like *(*Csl*)** Proteins

**DOI:** 10.3390/molecules26144335

**Published:** 2021-07-17

**Authors:** Gerasimos Daras, Dimitris Templalexis, Fengoula Avgeri, Dikran Tsitsekian, Konstantina Karamanou, Stamatis Rigas

**Affiliations:** Department of Biotechnology, Agricultural University of Athens, 11855 Athens, Greece; gdaras@aua.gr (G.D.); dimitempl@aua.gr (D.T.); favgeri@aua.gr (F.A.); dtsitsekian@aua.gr (D.T.); kon.karamanou@gmail.com (K.K.)

**Keywords:** cell wall, *Cellulose synthase*, cellulose biosynthesis inhibitors, CesA, Csl, 3D modeling, antimorphic mutations, dominant mutants

## Abstract

The wall is the last frontier of a plant cell involved in modulating growth, development and defense against biotic stresses. Cellulose and additional polysaccharides of plant cell walls are the most abundant biopolymers on earth, having increased in economic value and thereby attracted significant interest in biotechnology. Cellulose biosynthesis constitutes a highly complicated process relying on the formation of cellulose synthase complexes. *Cellulose synthase* (*CesA*) and *Cellulose synthase-like* (*Csl*) genes encode enzymes that synthesize cellulose and most hemicellulosic polysaccharides. *Arabidopsis* and rice are invaluable genetic models and reliable representatives of land plants to comprehend cell wall synthesis. During the past two decades, enormous research progress has been made to understand the mechanisms of cellulose synthesis and construction of the plant cell wall. A plethora of *cesa* and *csl* mutants have been characterized, providing functional insights into individual protein isoforms. Recent structural studies have uncovered the mode of CesA assembly and the dynamics of cellulose production. Genetics and structural biology have generated new knowledge and have accelerated the pace of discovery in this field, ultimately opening perspectives towards cellulose synthesis manipulation. This review provides an overview of the major breakthroughs gathering previous and recent genetic and structural advancements, focusing on the function of CesA and Csl catalytic domain in plants.

## 1. Introduction

The cell wall is the most significant determinant of plant shape with cardinal roles in plant development and growth. Cell walls are highly complex and show diverse chemical compositions throughout the plant species and can be modified dynamically during cell expansion and differentiation [1]. Their composition also depends on the cell type, developmental stage and environmental conditions [2]. Walls maintain continuity with plasma membrane and the cytoskeleton, and therefore control inner and outer communication. Plant defense against biotic stresses primarily relies on the cell wall, which provides the frontline of plant immunity [3]. Hence, the cell wall is crucial for plants to adapt and survive.

Plants form two types of cell wall which differ in respect to composition and function. The primary cell wall occurs when cells divide and elongate consisting of cellulose, hemicellulose and pectin. The secondary cell wall mostly consists of cellulose and is deposited as a thick layer between the primary cell wall and the plasma membrane after cessation of cell expansion [4]. Both types of plant cell wall are composed of polysaccharides. Cellulose is the major structural component, which is synthesized at the plasma membrane, while pectins and hemicelluloses are assembled in the Golgi apparatus. Cellulose and additional polysaccharides of plant cell walls represent the most abundant biopolymers on earth, providing important materials for industrial applications, construction, textile manufacture and biofuel production. Consequently, cell wall biology has drawn significant attention over the past decades and cellulose research represents an important field in plant biology.

*Cellulose synthase* (*CesA*) and *Cellulose synthase-like* (*Csl*) genes encode for glycosyltransferases type-2 (GT-2) enzymes that synthesize the *β*-1,4-linked glycan backbones of cellulose and most hemicellulosic polysaccharides, respectively. Cellulose is synthesized by the cellulose synthase complexes (CSCs) comprising six rosette subunits formed by multiple isoforms of CesA enzymes. CesA catalyze the assembly of *β*-1,4 glucan polymers in each rosette subunit forming protofibrils, which are subsequently transformed into cellulose microfibrils [5]. Cellulose synthases were initially identified in *Acetobacter xylinum*, whereas the plant cellulose synthase genes were first cloned and characterized in cotton [6]. Nevertheless, the primary study of cellulose synthase in *Arabidopsis thaliana* using forward genetics was a milestone, paving the way towards the analysis of *cesa* mutants [7]. Cellulose synthase-like (Csl) enzymes synthesize the non-cellulosic polysaccharide components of the cell wall and constitute a multigene family organized in distinct classes of proteins sharing sequence similarity with the CesA family. While many *Csl* genes have been characterized in various plant species, the specificity in terms of distinct types of polysaccharide production remains yet unclear.

Among plant species, *Arabidopsis* is an invaluable genetic model for the dissection of cell wall synthesis and thus can be representative of many higher dicotyledonous plants. Concerning monocotyledonous species, rice is an important model and a major commercial crop worldwide. Herein, we attempt to summarize current advances on cell wall construction through a comprehension of cellulose and non-cellulose synthesis based on these two models. This review particularly analyzes recent ground-breaking findings of genetics and structural studies on the catalytic domains of CesA and Csl in *Arabidopsis* and rice.

## 2. Classification of Plant *Cellulose synthase* and *Cellulose synthase-like* Proteins

**Figure 1:**
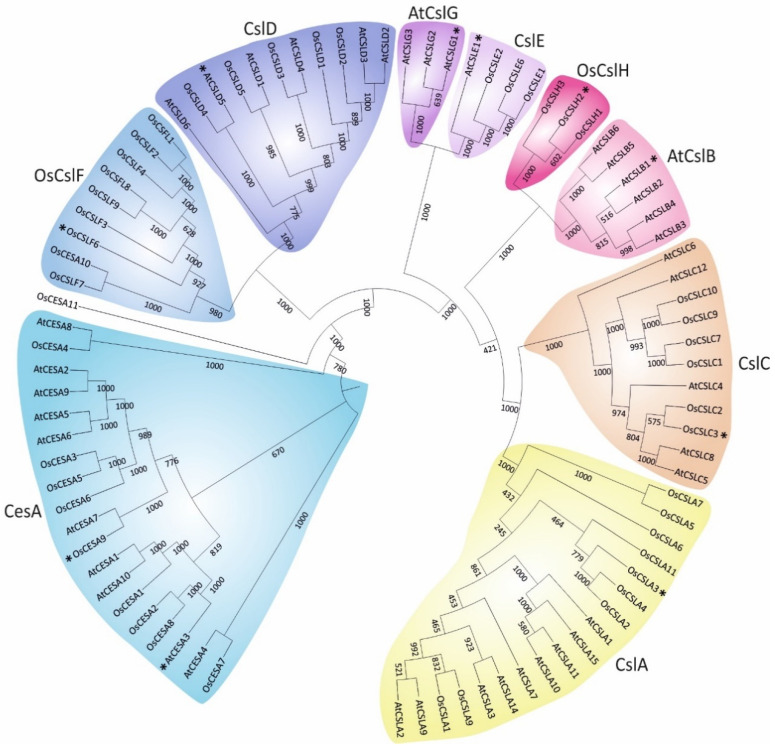
Circular phylogenetic tree of *Arabidopsis* (At) and rice (Os) CesA and Csl proteins. The neighbor-joining (NJ) tree was constructed using ClustalX2 and displayed by iTOL: https://itol.embl.de/ (accessed on 5 May 2021). The bootstrap values of the analysis based on 1000 replicates are presented. The asterisks (*) indicate the proteins used for 3D modeling. The accession numbers of *Arabidopsis* and rice CesA and Csl proteins are reported in Table S1.

Cell walls share common polysaccharide constituents across the plant kingdom and between distant species. Phylogenetic analysis of *Arabidopsis thaliana* (*At*) and rice *Oryza sativa* (*Os*) classified the CesA and Csl proteins in two major branches (Figure 1 and Table S1). All CesA proteins of both organisms were grouped in a single hyper branch, whereas the Csl branch was further divided into eight classes (CslA, CslB, CslC, CslD, CslE, CslF, CslG and CslH). This classification coincides with previous reports demonstrating that Csl proteins are Glycosyltransferase type-2 (GT2) enzymes, showing sequence similarity to CesAs [8,9]. As was anticipated, members of the CslJ/M class are not present in the genome of both *Arabidopsis* and rice, but exist in a few monocot and eudicot species [9,10]. An interesting point is that the paralogous proteins from the two species were grouped in different classes, in contrast to the orthologous, suggesting that CesA and Csl diverged before the split between dicots and monocots. Moreover, several proteins were grouped in pairs indicative of a common gene duplication event.

To obtain a deeper understanding of the origin and divergence of the CesA and Csl proteins, a genome-wide analysis of nine fully sequenced genomes was conducted (Table S2). Given the enormous diversity in architecture and life strategies, the analysis was restricted to vascular angiosperms (flowering plants) that evolved large aerial organs and acquired mechanisms for strengthening the plant body against the force of gravity and against an array of mechanical forces not encountered by algae and most bryophytes [11,12,13]. Moreover, based on previous genome-wide approaches, the algal CesA sequences were excluded because they were an outgroup from angiosperms, forming independent evolutionary clades [10,14]. Our analysis of four monocot and five dicot genomes confirmed that Csl proteins have a different evolutionary origin than CesA and showed that CslA and CslC classes deviate from the rest of the Csl proteins (Figure S1 and Table S2).

The *Arabidopsis* family of CesA proteins consists of ten members (Figure 1 and Table S1). CesA1, CesA3 and CesA6 are components of the primary wall cellulose synthase complex, whereas CesA4, CesA7 and CesA8 participate in the secondary wall cellulose synthesis [15,16,17,18]. CesA2, CesA5 and CesA9 constitute the CesA6-like proteins, which are partially redundant and interchangeable in the assembly of the cellulose synthase complex [17]. Furthermore, the function of CesA10 in cellulose synthesis is still not clear [19]. Even though CesA proteins are classified into two distinct major groups based on the type of cell wall production and their adaptive involvement in cellulose synthesis, they may preserve a dual mode of function. Interestingly, when *CesA1*, a primary cell wall gene, was expressed under the control of *CesA7* gene promoter that is involved in secondary cell wall synthesis, the secondary cell wall defects of the *Arabidopsis cesa8* knockout mutant were partially restored [20]. Inversely, when *CesA7* was driven by the promoter of *CesA3* primary cell wall gene, the growth phenotypic defects of *Arabidopsis cesa3* mutant were also partially restored [20]. *CesA9* has a nonredundant role in secondary cell wall synthesis, albeit on that has been shown to act synergistically with *CesA1* and *CesA3* in pollen and anthers [16,21]. Therefore, CesA proteins possibly share dynamic overlapping functions at particular developmental stages.

In rice, the family of CesA proteins consists of eleven members (Figure 1 and Table S1). However, rice CesA10 and CesA11 isoforms differentiate from the other members. CesA10 is relatively shorter and possibly represents a truncated protein isoform, whereas both CesA10 and CesA11 harbor only the typical cellulose synthase domain [22]. Interestingly, although rice CesA10 is classified as a putative cellulose synthase member, it is located within the CslF clade and shows a high similarity with CslF7 (Figure 1 and Table S3A,B).

Unlike cellulose, the composition of hemicellulose between monocots and dicots is highly diverged, supporting the presence of specific Csl classes that exist only in monocots or dicots [23]. The CslB and CslG classes seem to be unique to dicots and thus are not present in rice, and generally in cereals (Figure 1 and Table S1). Likewise, CslF and CslH classes were found exclusively in monocots [24,25]. The rest of the Csl classes are present in both monocots and dicots, indicative of their origin before the split in evolution [10]. In agreement with a previous report [26], the CslD class is closely related to CslF, showing the highest similarity with CesA (Figure 1). CslB is tightly clustered, with CslH forming a common clade as the CslG and CslE classes (Figure 1). Strikingly, CslA and CslC classes are highly diverged compared to CesA and the rest of Csl classes (Figure 1 and Table S3). This observation comes in agreement with the notion that CslA and CslC evolved from a common ancestral cellulose synthase gene of bacterial origin, while CesA and the remaining of Csl proteins emanated from a lateral transfer of a cyanobacterial cellulose synthase gene harboring a plant-conserved region (PCR) [27].

Based on experimental evidence, the Csl enzymes are widely appreciated to be involved in the synthesis of hemicellulose backbones. The CslA class consists of mannan synthase enzymes, which polymerize the 1,4-β-linked back bone of mannans and glucomannans [28,29]. The CslC class includes enzymes involved in 1,4-β-glucan back bone of xyloglucans or other polysaccharides [30,31]. The CslD enzymes are involved in synthesis of xylan and homogalacturonan, together with cellulose or mannan in tip-growing cells [32,33,34]. Recent reports showed that a chimeric fusion between CesA6 and the catalytic domain of CslD3 was able to complement the cell wall defects of the *Arabidopsis cesa6* mutant. Although CslD3 has a *β*-1,4-glucan synthase activity, these results support that CslD enzymes have novel roles in cellulose production [34]. The classes of CslF, CslH and CslJ enzymes participate in the synthesis of the mixed-linkage glucan [9,35,36,37] (MLG). Contrary to the rest of Csl members, the function of CslB/E/G/M classes is poorly characterized to date.

## 3. Structural Domains of *Cellulose synthase* and *Cellulose synthase-like* Proteins

**Figure 2:**
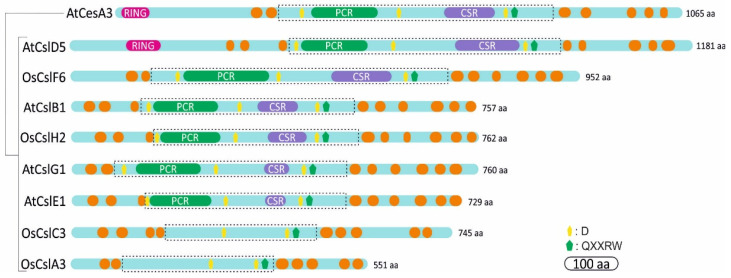
Domain structures of representative members of *Arabidopsis* (At) or rice (Os) CesA and Csl classes. The transmembrane (TM) helices are shown as orange rectangles. The N terminal region of CesA and CslD contains a RING type zinc finger domain. The plant-conserved region (PCR) and the class-specific region (CSR) are absent from CslA and CslC classes. The hydrophilic catalytic domain is boxed in a dotted line rectangle. The transmembrane regions were predicted using the PredictProtein server: https://predictprotein.org/ (accessed on 5 May 2021). The coordinates of PCR and CSR were estimated through protein alignment between the CesA and Csl members as previously described [46].

Cellulose synthase genes encode ~110 kDa CesA polypeptides, whereas the Csl proteins are relatively shorter with a protein length that varies depending on the class (Figure 2). CesA proteins bear an N-terminal domain (NTD) including a RING-type zinc finger motif, a large cytosolic domain flanked by two and five transmembrane helices [38] and a carboxyl terminal domain predicted to locate in the apoplast (Figure 2) [38,39]. The NTD is cytosolic and is based on the cotton CesA1; it modulates in vitro dimerization through zinc ion binding [40]. Further observations showed that NTD binds to Cellulose synthase-interactive-1-coated beads, indicating the significant role of the CesA N-terminus in protein–protein interactions [38]. The glycosyltransferase (GT) domain catalyzes the polymerization of glucan, using UDP-activated glucose as substrate, and lies in the cytoplasmic region including the triple conserved aspartic acids (D,D,D) and the QXXRW motif, which are common in all GT2 enzymes (Figure 2). The first two aspartic acid residues coordinate the UDP of the UDP-glucose, whereas the third aspartic acid serves as the catalytic base. The arginine (R) of the QXXRW motif also coordinates UDP, whereas tryptophan (W) forms Van der Waals interactions with the terminal glucan residues of the acceptor chain [41].

Plant CesA proteins contain a plant-conserved region (PCR) between the first two aspartic acid residues and a class-specific region (CSR) inside the large central cytoplasmic domain (Figure 2) [6]. The CSR is highly variable between the different CesA paralogues, whereas it is conserved among CesA homologs of different species. Presumably, the plant-conserved and class-specific regions mediate functions unique to plants. They may facilitate the localization of CesA to the membranes, multimerization and assembly of the CSC, interactions with other regulatory elements and other specific features of cellulose synthesis [42]. Intriguingly, domain swapping in *Arabidopsis* has shown that CSR is functionally interchangeable within different CesA members, resulting in partial complementation of *cesa* mutants [42,43,44]. Thus, CesA class specificity is independent of the CSR region. Moreover, the CSR is implicated in OsCesA8 dimerization and the PCR is involved in interactions between dimers [45]. Conversely, structural modeling predicted that the PCR of *Arabidopsis* CesA1 forms the base of the catalytic trimer outward to the cytosol [46]. Hence, the PCR region could recruit auxiliary non-CesA proteins, whereas CSR most likely is implicated in trimer–trimer interactions within the CSC.

Based on in silico prediction models, the catalytic domain of Csl resides in a large central cytosolic domain between multiple transmembrane helices, which differ in number depending on the Csl class (Figure 2). Interestingly, the RING-type zinc finger motif exists only at the N-terminus of CslD proteins, which show the highest similarity with CesA (Table S3). Among the Csl proteins, the members of CslA and CslC classes are short in terms of length and highly diverged, lacking the structural domains, which characterize the CesA proteins, namely the PCR and CSR regions.

## 4. The Architecture of CesA Catalytic Domain

Pioneering studies on the crystal structure of *Rhodobacter sphaeroides* BcsA-B complex described the catalysis of glucose to the nascent glucan chain, while translocating through the transmembrane (TM) pore into the apoplast. This work also clarified the role of the classical conserved residues D, D, D, QXXRW and additional functional motifs responsible for donor and acceptor binding [41]. Following these findings, protein structure predictions and molecular dynamics of cotton CesA1 demonstrated a catalytic fold that includes the conserved aspartic acids (D) and the QXXRW motif [47]. Apparently, the first two aspartic acid (D) residues coordinate Uridine diphosphate (UDP) of glucose and the essential divalent cation, while the third D might act as the catalytic base. The QXXRW motif resides in the interface helix 2 (IF2) interacting with the cellulose acceptor substrate [47]. Additional studies on the structure of the catalytic domain of *Arabidopsis* CesA1 provided the first experimental evidence for assembly of CesA into a stable homotrimer in solution [46], in contrast to previous studies proposing the formation of CesA homodimers [45]. Further modeling demonstrated that the CSR projects radially outward and does not participate in any interfaces within the trimer, whereas the PCR domain is essential for the formation of the catalytic trimer pointing toward the cytosol [46].

**Figure 3:**
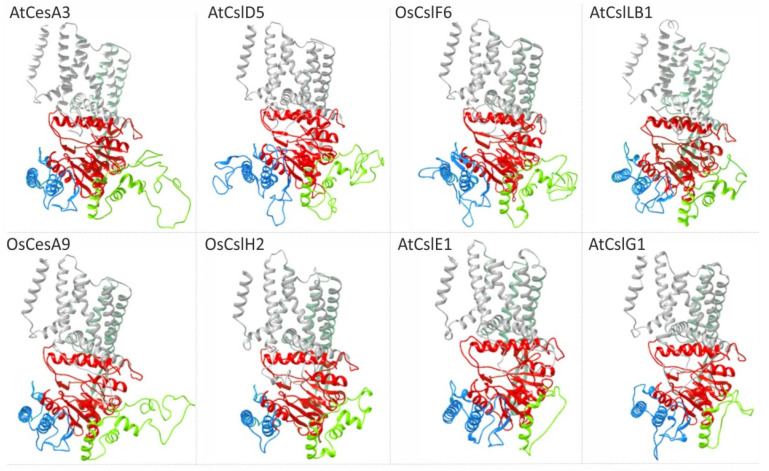
Structural 3D models of representative CesA and Csl members. Transmembrane (TM) domains are colored in grey and the catalytic domain is colored in red. PCR and CSR are presented with blue and green color, respectively.

**Figure 4:**
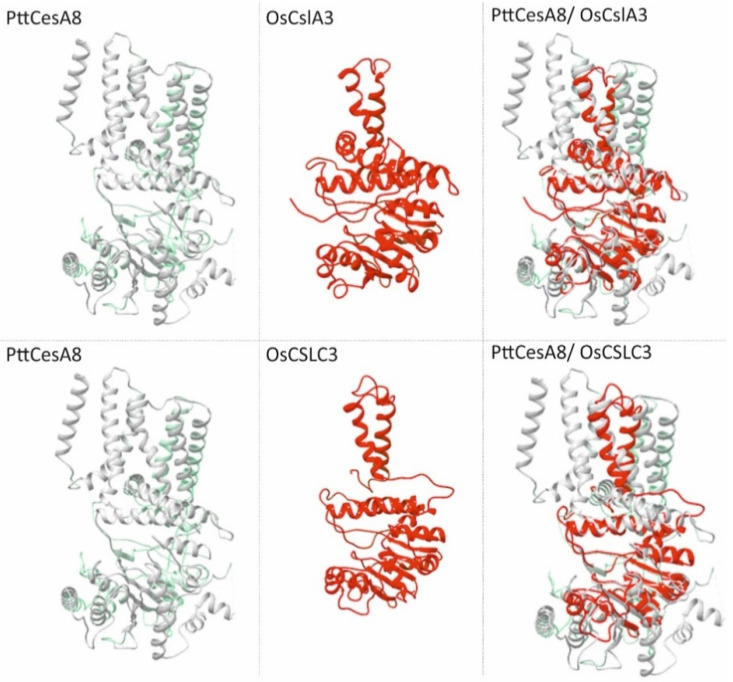
The structure of rice CslA and CslC is highly similar to the catalytic domain of poplar CesA8 (6wlb.1.A). Structural 3D model of PttCesA8 (left panel) and OsCslA3/C3 (middle panel). Superimposed structures of OsCslA3/C3 match only with the core catalytic domain and two transmembrane helices of PttCesA8 (right panel). The protein structure model of OsCslA3/C3 was based on the crystal structure of *Rhodobacter sphaeroides* BcsA (4p02.1.A).

Recent studies using cryo-electron microscopy (cryo-EM) determined the structure of the homotrimeric complex of poplar CesA8, which is associated with cellulose glucan chains [38]. The cytosolic domain was flanked by two and five TM helices at the N- and C-termini, respectively, whereas TM helices 1–6 cluster around three cytosolic interface helices (IF1-3) in agreement with previous studies describing the BcsA structure of *Rhodobacter* [38,41]. In addition, the intracellular catalytic domain of poplar CesA8, and generally of the plant CesA, consists of a 7-stranded β-sheet surrounded by six α-helices creating an active site pocket for substrate and acceptor binding (Figure 3 and Figure 4). The PCR of poplar CesA8 contains two anti-parallel α-helices connected by a linker of approximately twenty residues, while the CSR forms helical and disordered regions [38]. It was also showed that the homotrimeric complex of poplar CesA8 was stabilized by the cytosolic PCR and by a helical exchange within the transmembrane localized regions.

Another recent ground-breaking work revealed structural insights of the homotrimeric assembly of CesA7 from *Gossypium hirsutum* [48]. This study described the cryo-EM structure of homotrimeric CesA7 at 3.5-angstrom resolution with a symmetrical assembly similar to the structure of poplar CesA8. Furthermore, the homotrimer of cotton CesA7 was stabilized by the transmembrane helix 7 (TM7) and the PCR domains, whereas the CSR is located at the periphery of the catalytic domain, suggesting an involvement in the interaction with other rosette units or non-CesA auxiliary proteins [48]. Additionally, the crystal structure of *Arabidopsis* CesA3 catalytic domain with its substrate UDP-glucose provided new insights into the mechanism of cellulose synthesis [49]. This work revealed the structural basis of how the substrate UDP-Glucose and a metal ion Mn^2+^ which is required for cellulose synthesis, are coordinated in plant CesA complexes. In agreement with previous reports, the crystal structure of *Arabidopsis* CesA3 catalytic domain was divided into the core domain, the PCR and the CSR domain. The core catalytic domain, including the catalytic sites, was composed of a *β*-sheet with eight strands, flanked by two *α*-helices on one side and two *α*-helices with three *β*-strands on the other side [49]. An insertion of a cellulose binding helix towards the catalytic region was proposed without forming a strong interaction with the rest of the catalytic domain, proposing that it is flexible in the absence of cellulose and transmembrane helices [49]. The plant-specific domains, namely PCR and CSR, were clearly resolved, forming a sandwich with the core catalytic domain as previously described. The catalytic domain of *Arabidopsis* CesA3 could form a homodimer via residues located at the periphery of its core. These residues are highly conserved among the *Arabidopsis* CesA proteins, suggesting a common dimerization mode.

## 5. Structural Insights into Csl Catalytic Domain

Given that data regarding the structure of Csl proteins are not available as yet, compared to the pile of mostly recent information regarding the architecture of CesA, a comparative analysis was performed to identify the features of the Csl catalytic domain. To accomplish this task, the protein structure homology-modeling server SWISS-MODEL was used to search first for evolutionary related structures with BLAST and HHblits [50]. Representative members of *Arabidopsis* and rice CesA and Csl classes were selected (Figure 3 and Figure 4). As expected, the primary and secondary cell wall CesA proteins matched the target sequence of the recently analyzed poplar CesA8 [38]. Notably, members of CslB, CslD, CslE, CslF, CslG and CslH classes that share a relatively high degree of amino acid similarity with CesA, especially at the catalytic domain, also resulted in a match with poplar CesA8 (Figure 3 and Table S3). Strikingly, prediction analysis showed that 3D models of CslB/D/E/F/G/H display structural congruence, having analogous properties with the experimental elucidation of plant CesA structure [38,48] (Figure 3). In particular, both CesA and Csl formed a channel of transmembrane helices. The catalytic core of the glyosyltransferase (GT) domain faced the entrance of the transmembrane channel and the PCR domain located at the periphery of the GT domain. PCR and CSR domains were connected at the catalytic domain via long flexible loops projecting from both sides of the catalytic domain at a distance from the channel of TM helices. CSR seemed to be intrinsically disordered and flexible due to the high variability between the protein members.

The results of the predicted 3D model analysis were consistent with the experimental data of cellulose synthase complex structure [38,48], supporting the accuracy of the analysis. The 3D model prediction confirmed that in terms of structure, the catalytic domain between the CslA and CslC classes was highly similar, in agreement with the Csl classification which showed a close relation between these classes (Figure 1 and Figure 4). However, it was really surprising that the evolutionary distant members of CslA and CslC classes, which in terms of the primary structure shared a low-level similarity with CesA (Figure 1 and Table S3), matched perfectly with the core catalytic domain and two transmembrane helices of poplar CesA8 (Figure 4). This structural similarity supports the notion that even though CslA and CslC emanate from a common ancestral bacterial cellulose synthase counterpart [41], they probably represent primitive forms of CesA proteins.

## 6. The *cesa* Mutants within the Catalytic Domain Shed Light on Cellulose Synthesis

**Figure 5:**
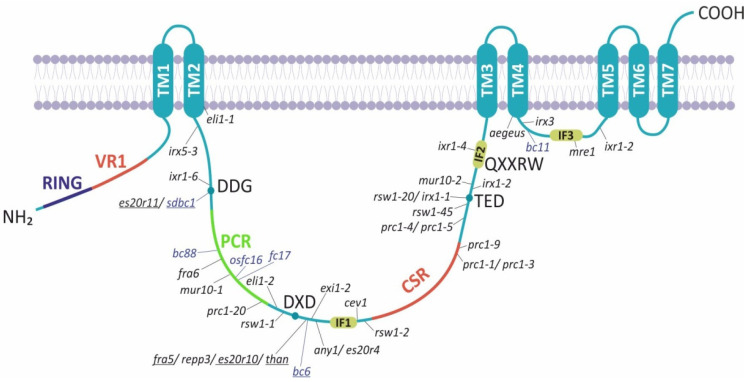
Schematic representation of CesA protein features. Previously characterized mutations in the catalytic and short hydrophilic domains of *Arabidopsis* and rice *CesA* genes are indicated in black and blue, respectively. The antimorphic mutations in the catalytic domain are underlined. The sites of mutations are shown relative to domains rather than by precise residue number. The transmembrane regions (TMs) and the orientation of the cytoplasmic domains were predicted using the PredictProtein software: https://predictprotein.org/ (accessed on 5 May 2021). VR1: Variable region-1; PCR: Plant-conserved region; CSR: Class-specific region; IF: Interphase helix.

Null mutations in essential genes like *CesA*, are typically lethal during embryogenesis or early seedling development. While a lethal phenotype due to catastrophic disruption of the cell wall complicates the strategies to elucidate cellulose biosynthesis, hypomorphic alleles that cause a partial loss of gene function have an important role on the genetic investigation of cell wall construction. As such, forward genetics screens were used to identify *cesa* mutants with developmental defects or altered responses to cellulose synthesis inhibitors (CBIs). Up to date, numerous mutant alleles of *CesA* in *Arabidopsis* and rice have been characterized and have been extensively investigated (Table S4). *CesA* point mutations have been identified throughout the polypeptide, including the catalytic domain. However, the point mutations mapped within the CSR are significantly rare, except for two alleles, *prc1-4/1-5* and *prc1-1/3*, residing at the end of the domain corresponding to nonsense mutations (Figure 5 and Table S4).

The *rsw1-1* mutation, which causes a radial swelling phenotype, was the first *cesa* mutant characterized in *Arabidopsis* [7]. The point mutation of *rsw1-1* was mapped between the first two aspartic acid signature residues in the catalytic domain of *Arabidopsis CesA1* gene (Figure 5). The *rsw1-1* mutant seedlings showed fifty percent reduction in cellulose production only when grown at an elevated temperature (31 °C). Furthermore, under restrictive temperature the *rsw1-1* mutation accumulated a non-crystalline form of *β*-1,4-glucan due to the disassembly of cellulose synthase complexes. The *rsw1-1* conditional swollen phenotype is typical of most primary cell wall *cesa* mutants and reminiscent of the effect of cellulose biosynthesis inhibitors on wild-type plants. A possible explanation may rely on the defects of cellulose synthase activity resulting in decreased anisotropic reinforcement, while cells retain their relative dimensions as they increase in volume. Recent studies on *rsw1-1* supported this notion demonstrating that perturbation of cell wall biosynthesis affects auxin transport and homeostasis, leading to defective anisotropic and isotropic growth of root cells [51]. It is widely speculated that the *rsw1-1* missense mutation could have broad effects on the overall plant structure and might destabilize CSC assembly under high temperatures [52]. An additional mutation residing in the catalytic domain of *Arabidopsis CesA1*, *rsw1-2*, exhibits a more drastic phenotype. The *RSW1-2* coding region bears a single nucleotide substitution causing a glycine to serine change at position 631 of CesA1 polypeptide (Figure 5 and Table S4). Despite the reduced cellulose levels, the *rsw1-2* allele displayed severe radial swelling of the root and hypocotyl cells at non-permissive temperatures [53].

An abnormal swollen cell phenotype was also evident in *rsw1-20* mutant, where the third aspartic acid residue the conserved glycosyltransferase motif “D, D, D, QXXRW” is changed to asparagine (Figure 5 and Table S4). This amino acid substitution is predicted by structural models to potentially abolish the catalytic activity of the *Arabidopsis* CesA1 enzyme due to a protein secondary structure rearrangement within residues 779–785 due to disruption of an *α*-helix [54]. Seedlings carrying the *rsw1-20* mutation appeared dwarfed with abnormal swollen cells in the cotyledons and the hypocotyl, whereas growth of the primary root was diminished leading to seedling lethality [54]. In contrast, though adjacent to *rsw1-20* allele, the *rsw1-45* mutant allele of *Arabidopsis* CesA1 caused by a substitution of the glutamic acid to lysine at position 779, showed less severe phenotypes, highlighting the specific role of the third aspartic acid residue of the conserved motif in the catalytic activity of CesA proteins [54]. Furthermore, *anisotropy1* (*any1*) is an additional missense mutant allele of *Arabidopsis* CesA1 with a dwarf phenotype, defective cell morphology and anisotropic growth of roots, aerial organs and trichomes [55]. Map-based cloning of *any1* revealed a single nucleotide change, resulting in amino acid substitution of the aspartic acid residue downwards of the DXD motif to asparagine in *Arabidopsis* CesA1 catalytic domain (Figure 5 and Table S4). Remarkably, *any1* displayed normal cellulose content, albeit both the cell wall crystallinity and velocity of CSCs was decreased.

Mutant alleles of *Arabidopsis CesA3* gene, namely *eli1-1/2* and *cev1*, carry missense mutations in the catalytic domain and show reduction at the cellulose levels, increased lignification and enhanced defense responses through overproduction of ethylene and jasmonate (Figure 5) [56,57]. The substitution of alanine 522 to valine in the *eli1*-2 lies within a flexible loop surrounding the substrate binding pocket [52] and thus *eli1*-2 mutation might result in faulty substrate acquisition. Moreover, *Arabidopsis* loss-of-function mutants of *CesA1* and *CesA3* genes have been reported as gametophytic lethal due to pollen defects suggesting the critical role of these core enzymes in cellulose biosynthesis [16].

Given that CesA6 is a component of primary cell wall CSC, the CesA2, CesA5, and CesA9, known as CESA6-like proteins, are partially redundant and interchangeable in the assembly of the CSC [17]. This is corroborated by the phylogeny of *Arabidopsis* CesA proteins, which shows high similarity between CesA6 and CesA6-like members (Figure 1 and Table S3). The *procuste1* (*prc1*) mutant of *CesA6* shows decreased cell elongation in roots and dark-grown hypocotyls due to cellulose deficiency and gapped walls [58]. Interestingly, the *prc-1-1* null mutant is viable, implying that its function is necessary for certain cell types or developmental stages and CesA6-like members might compensate for its activity in the CSC (Figure 5 and Table S4). Furthermore, recent findings demonstrated that mutation of the first conserved aspartic acid (D395N) in CesA6, resulted in an interruption of its anticipated transport to Golgi, proposing a significant functional role of catalytic activity towards *CesA* trafficking [59].

Expectedly, *cesa* mutants result in altered structure and reduced amounts of cellulose. However, despite the defective cellulose content, the composition of pectin and hemicellulose is often changed in primary cell wall *cesa* mutants including *rsw1-1* [60], *prc1-1* [58] and *aegeus* [61]. The rationale for pectin and hemicellulose defects is that altered cellulose proportion or structure can trigger compensatory production of non-cellulosic wall components, highlighting the cell wall compositional dynamics. In addition, when cellulose biosynthesis is chemically or genetically distorted through either mutations in *CesA* or Cellulose-interacting proteins, microtubule organization is consequently disrupted indicating a tight association of cellulose synthase activity with microtubule cortical array organization [62,63,64].

In general, mutants of *CesA* involved in the secondary cell wall synthesis present abnormalities in vasculature tissues. A remarkable example in this context is *irx5-3* allele, bearing a point mutation in the catalytic domain of *Arabidopsis* CesA4, which shows an irregular xylem phenotype with thin cell walls due to the reduction of cellulose content [18] (Figure 5 and Table S4). These phenotypic defects are identical of *irx1-1/2* mutant alleles of *Arabidopsis CesA8* that also harbor single amino acid mutations in the catalytic domain [65] (Figure 5 and Table S4). Another mutant allele of *CesA8*, *fragile fiber 6* (*fra6*), showed dramatic reduction in the mechanical strength of mature inflorescence stems and cellulose amount [66]. The *fra6* mutation is recessive and results in a missense amino acid change (R362K) in the catalytic domain (Figure 5 and Table S4). Additionally, the *exi1-2* mutant of *CesA8* has vascular defects with collapsed xylem and small rosette leaves due to defective cell expansion [67]. Likewise, *mur10-1/2* point mutations in the catalytic domain of *CesA7* had collapsed xylem vessels and showed abnormal plant growth, hypocotyl strength and fertility [68]. Additionally, knockout mutants of secondary cell wall *CesA*, such as *irx5-4* (*cesa4*), *irx3-7* (*cesa7*) and *irx1-7* (*cesa8*) harboring T-DNA insertions, exhibit various phenotypes including dark green leaves and inflorescence stems, collapsed xylem vessels, reduced plant height and low cellulose content [42]. Ιn rice, the *fc17* mutant of CesA4 protein, the orthologue of *Arabidopsis* CesA8 involved in secondary cell wall synthesis, harbors an amino acid substitution (F426S) in the PCR (Figure 5 and Table S4). The *fc17* mutation affected plant growth accompanied by higher lodging resistance compared to the wild-type [69]. The mutant also shows enhancement of biomass saccharification efficiency and reduction of cellulose content with a compensatory increase in hemicelluloses and lignin content. Therefore, a conclusion drawn by these observations is that the PCR might be a promising target for cell wall modification for biotechnological applications in rice [69]. Another secondary cell wall mutant in rice is *brittle culm 88* (*bc88*), which exhibits a diversity of pleiotropic phenotypes, including brittle culm at the whole-plant growth stages and withered leaf tips at the seedling stage [70]. The mutated *BC88* gene harbors a point mutation leading to an amino acid change from proline to leucine at position 421 of rice CesA9, which is an orthologue of *Arabidopsis* CesA7 [70].

Complementary to the hydrophilic catalytic domain, evidence supports that an additional short hydrophilic loop within TM4 and TM5 including IF3 lies on the cytoplasm and might control substrate access, affecting CesA function [71,72]. An additional conserved FxVTxK motif lies next to IF3 and functions as a substrate-gating loop analogous to BcsA of *Rhodobacter* [41,71]. The conserved amino acids of this motif bind to UDP as the UDP-glucose substrate for polymerization is inserted into the active site [73]. In this region, various mutations affecting cellulose synthesis have been identified (Figure 5 and Table S4). Interestingly, *aegeus* mutation of alanine to valine at position 903 of *Arabidopsis* CesA1 displayed quinoxyphen resistance [61], whereas *ixr1-2* mutation of glycine 998 to aspartic acid of CesA3 conferred isoxaben resistance [74]. Furthermore, *aegeus* and *ixr1-2* mutants of CesA1 and CesA3, respectively, displayed changes to the ordered crystallization of glucan chains in the interior of cellulose microfibrils [61]. The *irregular xylem3* (*irx3*) mutant of *Arabidopsis* CesA7 resulted from a nonsense mutation in place of tryptophan at position 859. Xylem cell walls of *irx3* contained low levels of crystalline cellulose that lead to collapsed xylem cells [75,76]. The *multiple response expansion1* (*mre1*) mutant of *Arabidopsis* caused by a point mutation of glycine to glutamic acid in *CesA3* gene at position 916, represented a weak allele displaying less cellulose content and pleiotropic developmental effects [77]. Functional characterization of the rice mutant *brittle culm11* (*bc11*), displayed growth retardation and dramatically reduced plant strength. This mutant occurred due to a missense mutation in rice CesA4 at position 858, with an amino acid substitution of glycine to arginine. The *bc11* mutant plants have aberrant secondary cell walls attributed to a significantly reduced cellulose content and abnormal secondary cell wall structure [78].

## 7. Antimorphic Mutations in the Catalytic Domain of CesA Proteins

In 1932, Hermann Joseph Muller, a geneticist and Nobel laureate, was the first to suggest a detailed classification of mutations, although at that time the molecular nature of a mutant was unknown [79]. Muller’s classification included among others the term “antimorphic mutation” to describe genetic alterations with a poisonous effect or an antagonistic interaction with a wild-type variant. An antimorphic mutation results in a structurally altered protein subunit, which is incorporated in a protein complex and renders the complex inactive. Remarkably, the severity of the phenotype depends on the dosage of the mutated over wild-type protein subunits incorporated in the complex.

**Figure 6:**
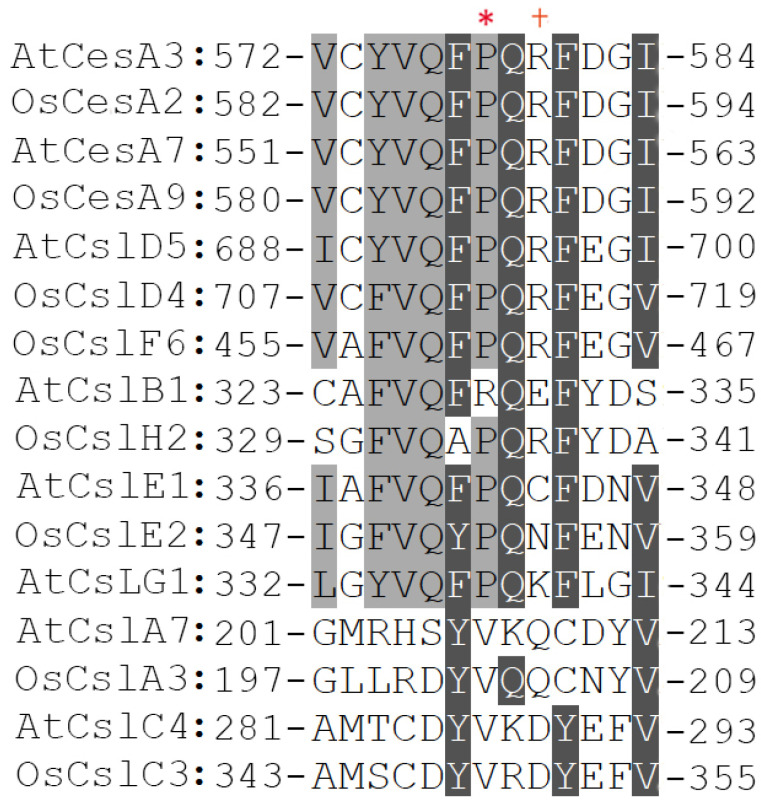
Multiple sequence alignment of *CesA* and Csl representative members reveals high evolutionary conservation of a set of amino acid residues within the catalytic domain. Among these residues, proline-578 of *Arabidopsis* CesA3 presented with an asterisk (*) was included known to generate semi dominant mutants. The cross (+) marks the arginine residue at position 588 of OsCesA9, which is substituted to glycine in rice *bc6* semi dominant mutant.

The dominant mutants of *CesA* are typical paradigms of antimorphs. As such, a nonconditional semi-dominant mutant of *Arabidopsis CesA3* resulted at homozygous state in a seedling lethal phenotype, whereas heterozygous plants were dwarf with a radially swollen root phenotype [80]. Due to lethality, the mutation was named *thanatos* (*than*) after the name of the ancient Greek entity of death. The dominant negative effect of *than* mutation was clearly reflected in the cellulose content, as it was decreased showing gene-dosage dependency. Positional cloning revealed an amino acid replacement of proline to serine at position 578 in the catalytic domain (Figure 5 and Table S4). This amino acid residue is located downstream of the conserved DXD motif in the globular region that modulates substrate catalysis and binding. The same proline residue was mutated to leucine in another allele of *Arabidopsis CesA3*, which resulted in a pleiotropic phenotype showing low cellulose content, short roots, and small cotyledons or hypocotyls. This mutation was named *regulator of PIN polarity* (*repp3*), as the mutant had defects in the basal localization of PIN1 auxin efflux carrier [81]. Curiously, unlike the *than* allele, *repp3* was viable as a homozygote. However, *repp3* seedlings showed intense phenotypic heterogeneity, including reduced germination and distorted seedling development, resembling to a great extent the homozygous seedling lethal phenotype of *than* mutant. Like *than* allele, when the conserved proline residue in *Arabidopsis CesA7*, involved in secondary cell wall synthesis, was changed to threonine (P557T) due to a point missense mutation resulted in a semi-dominant mutant (Figure 5 and Figure 6, Table S4). The homozygous seedlings of *fra5* mutation showed reduction in fiber wall thickness and cellulose content, whereas these developmental defects were mild in heterozygous mutant plants [66].

In rice, two alleles of *CesA9,* which is a gene ortholog of *Arabidopsis CesA7*, have been characterized as semi-dominant mutants (Figure 1). Firstly, *brittle culm 6 *(*bc6*)** mutation was generated by a single amino acid substitution of arginine (R588) to glycine, which lies two amino acid residues downstream of *Arabidopsis* CesA3 proline (P578) in the middle region of the catalytic domain (Figure 5 and Figure 6, Table S4) [82]. In homozygous *bc6* plants, whose stems were easily broken upon bending, the content of cellulose was reduced by 38% and hemicellulose proportion was remarkably increased by 34%. The *brittle culm* phenotype was retained in *bc6* heterozygous seedings demonstrating the semi dominant nature of the mutation. Secondly, the *semi-dominant brittle culm 1* (*sdbc1*) mutant carries a substitution of aspartic acid (D387) to asparagine (Figure 5 and Table S4). The *sdbc1* homozygous plants were characterized by reduced cellulose content and decreased secondary cell wall thickness [83]. Interestingly, *sdbc1* heterozygous plants exhibited increased salt tolerance. This finding is consistent with previous genetic and physiological studies pinpointing the role of *cesa* mutants in response to biotic and abiotic stress conditions [56,84,85,86]. Remarkably, the *es20r11* missense mutation of the same aspartic acid (D396) to asparagine in *Arabidopsis* CesA6 also resulted in a semi dominant reduced sensitivity to endosidin20 (ES20), which is a chemical inhibitor of *CesA* activity and trafficking (Figure 5 and Table S4) [87].

The negative dominant mutant alleles within the catalytic domain of *Arabidopsis* and rice *CesA* proteins define an up-to-date unknown motif that includes conserved amino acid residues like proline or arginine (Figure 6). Upon mutagenesis this region generated a series of antimorphic mutants, namely *than*, *fra5* and *bc6*. However, evidence based on 3D modeling predictions demonstrated that proline at position 492 of cotton CesA1, the proline analog of *Arabidopsis* CesA3 at position 578, acts as a hinge point of the solvent-accessible P492-G518 loop, which lies between two *β*-sheets [47]. The tip of this loop more likely contacts the QVLRW motif and modulates its interaction with the forming *β*-1,4-glucan chain. Molecular dynamics simulations revealed that the motion of this loop can be modified by mutations, resulting in diminished interaction of the QVLRW motif with the cellulose chain [47]. In silico experimental assays exhibited remarkable effects on the structure particularly when this predicted hinge point was altered to other amino acid residues. Hence, these mutations could adversely affect catalysis and substrate binding. Considering these recent observations, the dominant negative effects of *CesA* antimorphic mutations are likely attributed to the incorporation of mutated protein isoforms into the CSC impairing the production of normal *β*-1,4-glucan chains. Collectively, the proximity surrounding the proline residue has a critical role in the structure and function of cellulose synthase complexes. As this proline is conserved among CesAs and in certain Csl proteins [80] (Figure 6), directed amino acid substitutions would be valuable tools to generate viable and semi-dominant mutants with significant phenotypes to study the uncharacterized features of these complexes in model plants and in agronomically important non-model species.

## 8. Cellulose Biosynthesis Inhibitors: Potent Tools to Dissect the Mechanisms of Cellulose Biosynthesis

Cellulose biosynthesis inhibitors (CBI) are valuable resources for the development of broad-spectrum herbicides useful for weed control. Unlike most known herbicides, field resistance has not been reported in CBI [88]. Hence, biotechnological engineering to produce CBI-resistant crop plants would be beneficial for modern agriculture. Moreover, CBI are widely used tools to dissect the complexity and dynamics of cellulose synthase mechanisms [88,89,90]. CBI are mainly classified in three major groups, depending on their effect on CSC rosette. The first group is comprised of compounds that clear CSC from the plasma membrane focal plane, including isoxaben, quinoxyphen, CESTRIN and Acetobixan. Unlike the first group, inhibitors of the second group, like 2,6-dichlorobenzonitrile (DCB) and indaziflam, increase *CesA* in the plasma membrane accompanying by reduction or arrest of CSC movement. The third group includes compounds that modify *CesA* trajectory to the plasma membrane.

Chemical genetics identified mutants resistant or tolerant against various CBI. Single amino acid substitutions, mostly in the transmembrane regions of *CesA*, have been reported to confer resistance to CBI, suggesting a critical role of these residues and their proximity to the binding site of the inhibitor [91]. These CBI-resistant mutant alleles often display reduction in cellulose crystallinity but they do not exhibit any other evident cellulose deficiency [61,91]. A possible explanation is that the structural changes of CBI binding sites most likely do not disrupt the cellulose synthase catalytic activity. There are only a few cases of residue modifications in the catalytic domain of *CesA* associated with resistance to CBI [91]. This could be attributed to topological association of these residues with the transmembrane regions, reflecting the complexity of the *CesA* protein structure and the perplexed mode of CBI binding to specific regions. The *ixr1-4* mutant in Arabidopsis CesA3 displayed moderate resistance to isoxaben containing an arginine to lysine substitution within the QXXRW motif (Figure 5 and Table S4). In addition, the *ixr1-6* mutant of *Arabidopsis* CesA3 showed low resistance to isoxaben due to a substitution of serine at position 377 to phenylalanine just upstream of the first conserved aspartic acid of the catalytic domain (Figure 5 and Table S4). These mutations possibly reside in regions of the catalytic domain suggested to associate with the transmembrane regions responsible for CBI binding.

Endosidin20 (ES20) constitutes a broad-spectrum plant growth inhibitor, which was identified by a chemical library screen, having a different mode of action compared to the rest CBI [87,92]. Under ES20 effect, *Arabidopsis* seedlings exhibit reduced root and hypocotyl growth and present swollen root cells, a phenotype reminiscent of the primary cell wall *cesa* mutants. ES20 was found to inhibit cellulose synthesis through targeting the core of the catalytic domain of CesA [39]. Mutant alleles *es20r11*, *es20r10* and *es20r4* of *Arabidopsis* CesA6, carrying missense mutations in the central catalytic domain, displayed reduced sensitivity to ES20 (Figure 5 and Table S4) [39]. Inhibition of *CesA* catalytic activity by ES20 results in decreased motility and reduced delivery of CSC rosettes to the plasma membrane [39]. In addition, ES20 affects CSC exit from Golgi, but it does not disturb CSC transport from the endoplasmic reticulum (ER) to the Golgi [93]. Therefore, CSC intracellular trafficking is affected by the ES20-mediated defective catalytic activity that exclusively occurs in mutations within the catalytic domain of *CesA* involved in primary cell wall synthesis [59,93]. This observation supports the notion that distinct mechanisms modulate the trafficking between primary and secondary cell wall *CesA* proteins [59]. Recently, the ES20-1 analog was isolated with stronger inhibitory effects on plant growth and cellulose biosynthesis than ES20 [94]. Molecular docking approaches using a modeled structure of Arabidopsis CesA6, revealed that both ES20 and ES20-1 might have additional target sites at the transmembrane regions of *CesA* beyond the catalytic site.

## 9. Conclusions and Future Perspectives

The cell wall determines cellular structure, mediates cell-to-cell communication and controls the overall plant growth. As the major plant cell wall component, cellulose is the most abundant polymer on earth and is valuable for numerous industrial purposes. During the past two decades, remarkable progress has been made towards the understanding of the mechanisms controlling cellulose synthesis or cell wall formation in plants. A plethora of *cesa* and *csl* mutants became important genetic tools uncovering functional insights of individual protein isoforms and their impact on plant development. The use of cellulose biosynthesis inhibitors together with chemical genetics dissected novel aspects of CSC catalytic mechanisms. There is no doubt that structural biology has recently pushed the frontiers of knowledge, highlighting the assembly mechanisms of *CesA* complexes and providing insights about the dynamics of cellulose production. Nevertheless, there are still many questions that need an answer to comprehend the elegance of cell wall heterogeneity and the existence of multiple cell wall enzyme homologs across plant species. Although *Arabidopsis* remains an invaluable model species, efforts are shifted progressively to non-model and commercially important species to tackle specific questions and accelerate biotechnological advancements. In the near future, multidisciplinary approaches are needed to highlight in-depth cell wall architecture and biophysics of cellulose synthesis. A better understanding of these phenomena, at both molecular and cellular levels, will have important ramifications in plant biotechnology and modern agriculture.

## Data Availability

No new data were created or analyzed in this study. Data sharing is not applicable to this article.

## Supplementary Materials

The following are available online, Table S1: Accession numbers and amino acid length of *Arabidopsis* and rice *CesA* and *Csl* proteins. Proteins selected for 3D modeling are presented in bold. Table S2: *CesA* and *Csl* proteins of representative angiosperms. Table S3: Amino acid sequence similarity between *CesA* and *Csl* proteins. (A) Percentage of exact matches between whole proteins. (B) Percentage of exact matches between the catalytic domain. Table S4: Inventory of *Arabidopsis* and rice cellulose synthase *cesa* mutant alleles. Figure S1: Unrooted phylogenetic tree of *CesA* and *Csl* proteins from rice (*Oryza sativa*), *Arabidopsis* (*Arabidopsis thaliana*), cucumber (*Cucumis sativus*), cotton (*Gossypium hirsutum*), pineapple (*Ananas comosus*), tomato (*Solanum lycopersicum*), maize (*Zea mays*), banana (*Musa acuminata*) and poplar (*Populus trichocarpa*).
