# Supplementary material for: Updating Insights into the Catalytic Domain Properties of Plant Cellulose synthase (CesA) and Cellulose synthase-like (Csl) Proteins
Source: Molecules. 2021 Jul 17;26(14):4335. doi: 10.3390/molecules26144335 (PMC8306620; doi:10.3390/molecules26144335)
Supplement: Supplementary file 1 [file molecules-26-04335-s001.zip › Table_S4.pdf]

Table S4. Inventory of *Arabidopsis* and rice cellulose synthase (*cesa*) mutant alleles

Gene	CesA	Allele	Mutation	Phenotype	Reference
At4g32410	*AtCESA1*	*rsw1-1*	A(549)V	Root swelling, stunted growth (heat, temperature sensitive)	[1]
		rsw1–20	D(780)N	Swollen root, abnormal dark morphology, reduced growth of hypocotyls and roots	[2]
		rsw1-45	E(779)K	Swollen root, abnormal dark morphology, reduced growth of hypocotyls and roots	[2]
		rsw1-2	G(631)S	Lethality	[3]
		aegeus	A(903)V	Quinoxyphen resistant, reduced cellulose content, altered cellulose crystallinity, increased CSC velocity	[4]
		any1	D(604)N	Dwarf plants with reduced anisotropic growth of roots, aerial organs, and trichomes	[5]
At5g05170	*AtCESA3*	*ixr1-2*	G(998)D	Semidominant resistance to isoxaben	[6]
		cev1	G(617)E	Stunted root growth, induction of defense responses	[7]
		eli1-1	S(301)F	Stunted root growth, induction of defense responses	[8]
		eli1-2	A(522)V	Stunted root growth, induction of defense responses	[8]
		than	P(578)S	Root swelling, stunted growth at permissive temperature, semidominant mutation, lethality	[9]
		mre1	G916E	Short, swollen root and etiolated hypocotyl, cellulose deficiency, dwarf plants	[10]
		ixr1-6	S(377)F	Isoxaben resistant, decreased cellulose content, cellulose crystallinity and plant height. Ectopic lignin deposition. Swollen roots.	[11,12] [12]
		ixr1-4		Isoxaben resistant, decreased cellulose content, cellulose crystallinity and plant height. Ectopic lignin deposition. Swollen roots.	
At5g44030	*AtCESA4*	*irx5-3*	Q(263)Stop	Irregular xylem vessels	[13]
At5g64740	*AtCESA6*	*prc1-1/1-3*	Q(720)Stop	Reduced growth of dark-grown hypocotyls and roots (many alleles), root hair henotype	[14,15]
		prc1-4/1-5	W(777)Stop	Reduced growth of dark-grown hypocotyls and roots	[15]
		prc1-9	K(722)S	Reduced growth of dark-grown hypocotyls and roots	[16]
		prc1-20		Oryzalin hypersensitive, isoxaben hypersensitive, short, swollen etiolated hypocotyls and roots, cellulose content deficiency	
		es20r4	D(605)N	Reduced sensitivity to Endosidin20, less long roots	[17]
		es20r10	P(595)S	Reduced sensitivity to Endosidin20, long roots	[17]
		es20r11	D(396)N	Reduced sensitivity to Endosidin20, long roots	[17]
At5g17420	*AtCESA7*	*fra5*	P(557)T	Fragile fibers, weak stem, extremely thin fiber walls, semidominant mutation	[18]
		mur10-1	W(444)Stop	Reduction in growth and dark-green coloration of the aerial parts	[19]
		mur10-2	H(734)Y	Reduction in growth and dark-green coloration of the aerial parts	[19]
		irx3	W(589)Stop	Irregular xylem. Low levels of crystalline cellulose and collapsed xylem cells	[20,21]
At4g18780	*CESA8*	*irx1-1*	D(683)N	Weak stem, collapsed xylem vessels	[22]
		irx1-2	S(679)L	Weak stem, collapsed xylem vessels	[22]
		fra6	R(362)K	Reduced fiber wall thickness	[18]
		exi1-2	G(508)E	Small rosette leaves, flowers, siliques, reduced cell elongation, dwarf plants, altered vasculature and cell morphology, cellulose content deficiency, impaired water transport	[23]

Gene	Cesa	Allele	Mutation	Phenotype	Reference
Os01g54620	*CESA4*	*bc11*	G(858)R	Reduced mechanical strength in the culms and leaves, abnormal growth, dwarfism, withered leaf tips	[24]
		fc17	F(426)S	Brittleness, reduced extension force but no significant reduction in breaking force of basal stem internode	[25]
Os09g25490	*CESA9*	*bc88*	P(421)L	Brittle phenotype with pleiotropic phenotypes, withered leaf tips	[26]
		bc6	R588G	Brittle phenotype but no pleiotropic phenotypes, culms and leaves easily broke	[27]
		osfc16	$W^{481}P^{482}GN \rightarrow C^{481}S^{482}GN$	Normal plant growth and high biomass production	[25]
		sdbc1	D387N	Mild brittle culm phenotypes	[28]

References

1. Arioli, T.; Peng, L.; Betzner, A. S.; Burn, J.; Wittke, W.; Herth, W.; Camilleri, C.; Höfte, H.; Plazinski, J.; Birch, R.; Cork, A.; Glover, J.; Redmond, J.;Williamson, R. E.. Molecular analysis of cellulose biosynthesis in Arabidopsis. *Science* **1998**, *279*, 717-720.

2. Beeckman, T.; Przemeck, G. K.; Stamatiou, G.; Lau, R.; Terryn, N.; De Rycke, R., Inzé, D,; Berleth, T.. Genetic complexity of cellulose synthase A gene function in Arabidopsis embryogenesis. *Plant Physiol* **2002**, *130*, 1883-1893.

3. Gillmor, C. S; Poindexter, P.; Lorieau, J.; Palcic, M. M.; Somerville, C. α-Glucosidase I is required for cellulose biosynthesis and morphogenesis in Arabidopsis. *J Cell Biol* **2002**, *156*, 1003-1013.

4. Harris, D. M.; Corbin, K.; Wang, T.; Gutierrez, R.; Bertolo, A. L.; Petti, C.; Smilgies, D. M.; Estevez, J. M.; Bonetta, D.; Urbanowicz, B. R.; Ehrhardt, D. W.; Somerville, C. R.; Rose, J. K. C.; Hong, M.; DeBolt, S. Cellulose microfibril crystallinity is reduced by mutating C-terminal transmembrane region residues CESA1A903V and CESA3T942I of cellulose synthase. *Proc Natl Acad Sciences USA* **2012**, *109*, 4098-4103.

5. Fujita, M.; Himmelspach, R.; Ward, J.; Whittington, A.; Hasenbein, N.; Liu, C.; Truong, T. T.; Galway, M. E.; Mansfield, S. D.; Hocart, C. H,; Wasteneys, G. O. The anisotropy1 D604N mutation in the Arabidopsis cellulose synthase1 catalytic domain reduces cell wall crystallinity and the velocity of cellulose synthase complexes. *Plant Physiol* **2013**, *162*, 74-85.

6. Scheible, W. R.; Eshed, R.; Richmond, T.; Delmer, D.; Somerville, C. Modifications of cellulose synthase confer resistance to isoxaben and thiazolidinone herbicides in Arabidopsis Ixr1 mutants. *Proc Natl Acad Sci USA* **2001**, *98*, 10079-10084.

7. Ellis, C.; Karafyllidis, I.; Wasternack, C.; Turner, J. G. The Arabidopsis mutant cev1 links cell wall signaling to jasmonate and ethylene responses. *Plant Cell* **2002**, *14*, 1557-1566.

8. Caño-Delgado, A.; Penfield, S.; Smith, C.; Catley, M.; Bevan, M. Reduced cellulose synthesis invokes lignification and defense responses in Arabidopsis thaliana. *Plant J* **2003**, *34*, 351-362.

9. Daras, G.; Rigas, S.; Penning, B.; Milioni, D.; McCann, M. C.; Carpita, N. C.; Fasseas, C.; Hatzopoulos, P. The thanatos mutation in Arabidopsis thaliana cellulose synthase 3 (AtCesA3) has a dominant-negative effect on cellulose synthesis and plant growth. *New Phytol* **2009**, *184*, 114-126.

10. Pysh, L.; Alexander, N.; Swatzyna, L.; Harbert, R. Four alleles of AtCESA3 form an allelic series with respect to root phenotype in Arabidopsis thaliana. *Physiol plant* **2012**, *144*, 369-381.

11. Sethaphong, L.; Davis, J. K.; Slabaugh, E.; Singh, A.; Haigler, C. H.; Yingling, Y. G. Prediction of the structures of the plant-specific regions of vascular plant cellulose synthases and correlated functional analysis. *Cellulose* **2016**, *23*, 145-161.

12. Shim, I.; Law, R.; Kileeg, Z.; Stronghill, P.; Northey, J. G.; Strap, J. L.; Bonetta, D. T. Alleles causing resistance to isoxaben and flupoxam highlight the significance of transmembrane domains for CESA protein function. *Front Plant Sci* **2018**, *9*, 1152.

13. Taylor, N. G.; Howells, R. M.; Huttly, A. K.; Vickers, K.; Turner, S. R. Interactions among three distinct CesA proteins essential for cellulose synthesis. *Proc Natl Acad Sci USA* **2003**, *100*, 1450-1455.

14. Desnos, T.; Orbovic, V.; Bellini, C.; Kronenberger, J.; Caboche, M.; Traas, J.; Hofte, H. Procuste1 mutants identify two distinct genetic pathways controlling hypocotyl cell elongation, respectively in dark-and light-grown Arabidopsis seedlings. *Development* **1996**, *122*, 683-693.

15. Fagard, M.; Desnos, T.; Desprez, T.; Goubet, F.; Refregier, G.; Mouille, G.; McCann, M.; Rayon, C.; Vernhettes, S.; Höfte, H. PROCUSTE1 encodes a cellulose synthase required for normal cell elongation specifically in roots and dark-grown hypocotyls of Arabidopsis. *Plant Cell* **2000**, *12*, 2409-2423.

16. Paredez, A. R.; Persson, S.; Ehrhardt, D. W.; Somerville, C. R. Genetic evidence that cellulose synthase activity influences microtubule cortical array organization. *Plant Physiol* **2008**, *147*, 1723-1734.

17. Huang, L.; Li, X.; Zhang, W.; Ung, N.; Liu, N.; Yin, X.; Li, Y.; Mcewan, R. E.; Dilkes, B.; Dai, M.; Hicks, G. R.; Raikhel, N. V.; Staiger, C. J.;Zhang, C. Endosidin20 targets the cellulose synthase catalytic domain to inhibit cellulose biosynthesis. *Plant Cell* **2020**, *32*, 2141-2157.

18. Zhong, R.; Morrison, W. H.; Freshour, G. D.; Hahn, M. G.; Ye, Z. H. Expression of a mutant form of cellulose synthase AtCesA7 causes dominant negative effect on cellulose biosynthesis. *Plant Physiol* **2003**, *132*, 786-795.

19. Bosca, S.; Barton, C. J.; Taylor, N. G.; Ryden, P.; Neumetzler, L.; Pauly, M.; Roberts, K.; Seifert, G. J. Interactions between MUR10/CesA7-dependent secondary cellulose biosynthesis and primary cell wall structure. *Plant physiol* **2006**, *142*, 1353-1363.

20. Taylor, N. G.; Scheible, W. R.; Cutler, S.; Somerville, C. R.; Turner, S. R. The irregular xylem3 locus of Arabidopsis encodes a cellulose synthase required for secondary cell wall synthesis. *Plant cell* **1999**, *11*, 769-779.

21. Ha, M. A.; MacKinnon, I. M.; Šturcová, A.; Apperley, D. C.; McCann, M. C.; Turner, S. R.; Jarvis, M. C. Structure of cellulose-deficient secondary cell walls from the irx3 mutant of Arabidopsis thaliana. *Phytochemistry* **2002**, *61*, 7-14.

22. Taylor, N. G.; Laurie, S.; Turner, S. R. Multiple cellulose synthase catalytic subunits are required for cellulose synthesis in Arabidopsis. *Plant Cell* **2000**, *12*, 2529-2539.

23. Chen, Z.; Hong, X.; Zhang, H.; Wang, Y.; Li, X.; Zhu, J. K.;Gong, Z. Disruption of the cellulose synthase gene, AtCesA8/IRX1, enhances drought and osmotic stress tolerance in Arabidopsis. *Plant J* **2005**, *43*, 273-283.

24. Zhang, B.; Deng, L.; Qian, Q.; Xiong, G.; Zeng, D.; Li, R.; Guo, L.; Li, J.; Zhou, Y. A missense mutation in the transmembrane domain of CESA4 affects protein abundance in the plasma membrane and results in abnormal cell wall biosynthesis in rice. *Plant Mol Biol* **2009**, *71*, 509-24.

25. Li, F.; Liu, S.; Xu, H.; Xu, Q. A novel FC17/CESA4 mutation causes increased biomass saccharification and lodging resistance by remodeling cell wall in rice. *Biotechnol Biofuels* **2018**, *11*, 1-13.

26. Rao, Y. C.; Yang, Y. L.; Xin, D. D.; Li, X. J.; Zhai, K. E.; Ma, B. J.; Pan, J. W.; Qian, Q.; Zeng, D. L. Characterization and cloning of a brittle culm mutant (bc88) in rice (Oryza sativa L.). *Sci Bull* **2013**, *58* , 3000-3006.

27. Kotake, T.; Aohara, T.; Hirano, K.; Sato, A.; Kaneko, Y.; Tsumuraya, Y.; Takatsuji, H.; Kawasaki, S. Rice Brittle culm 6 encodes a dominant-negative form of CesA protein that perturbs cellulose synthesis in secondary cell walls. *J Exp Bot* **2011**, *62*, 2053-2062.

28. Ye, Y.; Wang, S.; Wu, K.; Ren, Y.; Jiang, H.; Chen, J.; Tao, L.; Fu, X.; Liu, B.; Wu, Y. A Semi-Dominant Mutation in OsCESA9 Improves Salt Tolerance and Favors Field Straw Decay Traits by Altering Cell Wall Properties in Rice. *Rice (NY)* **2021**, *14*, 1-19.
