# Supplementary figures and images for: Updating Insights into the Catalytic Domain Properties of Plant Cellulose synthase (CesA) and Cellulose synthase-like (Csl) Proteins
Source: Molecules. 2021 Jul 17;26(14):4335. doi: 10.3390/molecules26144335 (PMC8306620; doi:10.3390/molecules26144335)

CsIF

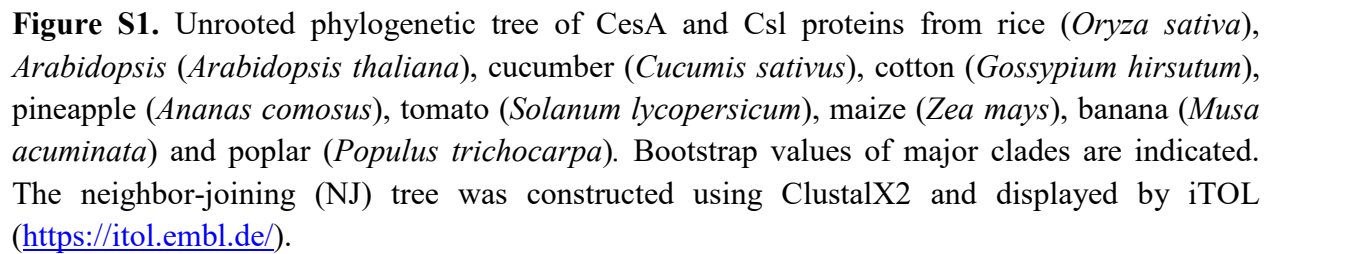

Supplement: Supplementary file 1 [file molecules-26-04335-s001.zip › Figure S1.pdf]
